# China Medical Missionary Association

**Published:** 1914-07-18

**Authors:** 


### CHINA MEDICAL MISSIONARY ASSOCIATION.

The biennial meeting of the China Medical Missionary Association will be held in Shanghai on Monday, February 1, 1915, and four following days. The last meeting of the Association, held in Peking, when the President of the Chinese Republic officially welcomed the Association, suggests that the forthcoming one will be equally well attended and profitable. It is estimated that at least a fourth of the four hundred to five hundred members will attend, and papers of a high order are expected. Papers to be read will be printed as abstracts, thus allowing time for more discussion; they should be in the hands of the programme committee by December 1, 1914. All business communications as to trade exhibits and all inquiries should be addressed in the first instance to Mr. Arthur F. Cole (on behalf of the programme committee), Ningpo, China.

